# The Hsp90 Inhibitor 17-DMAG Attenuates Hyperglycemia-Enhanced Hemorrhagic Transformation in Experimental Stroke

**DOI:** 10.1155/2021/6668442

**Published:** 2021-02-02

**Authors:** Jiaming Zhang, Kai Wang, Jia Qi, Xiaodong Cao, Feng Wang

**Affiliations:** ^1^Department of Emergency, Wuxi People's Hospital Affiliated to Nanjing Medical University, Wuxi, China 214023; ^2^Department of Neurology, Second Affiliated Hospital of Xuzhou Medical University, Xuzhou City, Jiangsu Province, China; ^3^Department of Pharmacy, Xinhua Hospital Affiliated to Shanghai Jiaotong University School of Medicine, Shanghai, China; ^4^Department of Neurology, Shanghai 7th Hospital Affiliated to Shanghai Traditional Chinese Medical University, Shanghai, China 200000

## Abstract

**Introduction:**

Hemorrhagic transformation (HT) is one of the most common complications of ischemic stroke which is exacerbated by hyperglycemia. Oxidative stress, inflammatory response, and matrix metalloproteinases (MMPs) have been evidenced to play a vital role in the pathophysiology of HT. Our previous study has reported that 17-DMAG, an Hsp90 inhibitor, protects the brain against ischemic injury via inhibiting inflammation and reducing MMP-9 after ischemia. However, whether 17-DMAG would attenuate HT in hyperglycemic middle cerebral artery occlusion (MCAO) rats is still unknown.

**Methods:**

Acute hyperglycemia was induced by an injection of 50% dextrose. Rats were pretreated with 17-DMAG before MCAO. Infarction volume, hemorrhagic volume neurological scores, expressions of inflammatory molecules and tight junction proteins, and activity of MMP-2 and MMP-9 were assessed 24 h after MCAO.

**Results:**

17-DMAG was found to reduce HT, improve neurological function, and inhibit expressions of inflammatory molecules and the activation of MMPs at 24 h after MCAO.

**Conclusion:**

These results implicated that Hsp90 could be a novel therapeutic target in HT following ischemic stroke.

## 1. Introduction

Hemorrhagic transformation (HT) occurs in 10–4% of patients suffering from ischemic stroke, leading to significant morbidity and mortality in affected individuals [1, 2]. Previous studies have clearly demonstrated that hyperglycemia can increase HT incidence following ischemic stroke, potentially leading to more serious brain infarction in these patients [3]. This hyperglycemia-mediated enhancement of HT is known to be associated with increased oxidative stress and inflammatory activity, potentially leading to blood-brain barrier (BBB) disruption and the death of neurons [4]. This BBB disruption is thought to be primarily mediated by matrix metalloproteinases (MMP-2 and MMP-9) that are produced in response to inflammation and oxidative stress, thereby leading to the development of HT in ischemic stroke patients [5]. However, at present, the specific molecular mechanisms governing the onset of HT remain poorly understood, and no effective treatments are available that are capable of preventing HT onset or curtailing HT-associated damage in patients after ischemic stroke.

The highly conserved chaperone protein Hsp90 (heat shock protein 90) is essential to guiding and stabilizing the folding of over 200 different proteins, including myriad transcription factors, kinases, and signaling regulators [6]. In the context of inflammation and oxidative stress as occurs during ischemic stroke, Hsp90 has further been shown to play an essential role in regulating the expression and activity of MMP proteins [7]. We have previously found that serum Hsp90 levels are significantly in patients following ischemic stroke and that these levels correlate with MMP-9 expression, thus raising the possibility that Hsp90 may play a key role in regulating MMP-9 expression and consequent disruption of the BBB after cerebral ischemia [8]. However, whether Hsp90 inhibition is effective for HT in hyperglycemia condition still requires further investigation. Several potent Hsp90 inhibitors have been developed to date, with some of these compounds having been tested in clinical trials aimed at exploring their utility as a means of treating cancer. The selective Hsp90 inhibitor 17-dimethyl-aminothylamino-17-demethoxy-geldanamycin (17-DMAG) has been found to function through its ability to block the Hsp90 ATP binding site, thereby facilitating the degradation of certain Hsp90 target proteins [9]. We have recently found that 17-DMAG is capable of decreasing oxidative stress and inflammation in mice, thereby leading to reduced MMP expression and decreased ischemic brain damage and abdominal aortic aneurysm-associated morbidity and mortality [7, 8]. As MMPs are known to be key mediators of hyperglycemia-enhanced HT development after ischemic stroke, we therefore hypothesized that inhibiting Hsp90 may represent an effective means of treating HT in this context. In the present study, we therefore tested the ability of 17-DMAG to suppress the development or severity of acute hyperglycemia-enhanced HT in an experimental stroke model and to explore the molecular mechanisms underlying such suppression.

## 2. Materials and Methods

### 2.1. Statement of Ethics

The research conforms to the Guide for the Care and Use of Laboratory Animals published by the US National Institutes of Health (NIH Publication No. 85-23, revised 1996), and the protocol was approved by the Institutional Animal Care Committee at Nanjing Medical University.

### 2.2. Middle Cerebral Artery Occlusion (MCAO) Model

Rats used for this study were randomized into three groups: sham, MCAO, or 17-DMAG+MCAO. The MCAO procedure was conducted in these rats as in past studies [10]. Briefly, a small incision was made in the external carotid artery through which a 4-0 nylon monofilament with a rounded tip was introduced. After 1.5 h, this suture was removed to permit the initiation of reperfusion. The surgical procedure in sham rats was identical, but the suture was not inserted. At 30 minutes prior to MCAO, rats in both the MCAO and 17-DMAG+MCAO groups were intraperitoneally injected with 50% dextrose (DX, 6 ml/kg) in order to induce acute hyperglycemia. Immediately following MCAO treatment, appropriate animals were intraperitoneally administered 17-DMAG (0.2 mg/kg) or vehicle control, with doses having been selected based on our previous analysis [8].

### 2.3. Hemorrhagic Volume Measurement

A spectrophotometric assay was used to assess the volume of cerebral hemorrhage in model animals, as in previous studies [11]. Briefly, a standard curve was generated using a mock-up hemorrhage model wherein specific volumes of blood were added to perfused brain tissue samples, after which hemispheric brain tissue underwent sonication-based homogenization. These homogenates were then spun for 30 minutes at 13,000 rpm, after which 400 *μ*l of the supernatant was collected and combined with 1.6 Drabkin reagent (Sigma). A spectrophotometer (Spectronics 3000; Milton-Roy) was then used to quantify sample absorbance at 540 nm. Hemoglobin content in experimental animals was determined through comparison with the resultant standard curve, thus allowing for quantification of hemorrhage volume.

### 2.4. 2,3,5-Triphenyltetrazolium Chloride (TTC) Staining

TTC staining was used to measure infarct volume, as in previous reports [12]. Briefly, 24 h post-MCAO, rats underwent deep anesthetization using isoflurane and were then euthanized. Brain tissue was collected and separated into six total serial coronal sections at 2 mm thickness. These sections were then added to 2% TTC for 15 minutes prior to analysis. The relative infarct size was calculated with the following equation: relative infarct size = (contralateral area − ipsilateral noninfarct area)/contralateral area.

### 2.5. Neurobehavioral Assessments

A previously developed scoring system was used to gauge neurological deficits in study animals at 24 h post-MCAO [13]. A researcher who was blinded to animal group assignments made all neurobehavioral measurements. Six individual test scores (spontaneous activity, symmetry in the movement of four limbs, forepaw outstretching, climbing, body proprioception, and response to vibrissal touch) were added together to yield an overall score that was between 3 and 18. The cumulative score for each group was then calculated.

### 2.6. Western Blotting

Cortical tissue samples were collected, and total protein was then extracted and quantified with a BCA kit (Thermo Scientific). Equivalent protein amounts then underwent 10% SDS-PAGE separation and were transferred to PVDF membranes for 2 h at 300 mA, and blots were then blocked with 5% nonfat milk in TBST for 1 h. Next, blots were probed overnight with antibodies specific for the following: histidine adduct (1 : 1000; Abcam); nitrotyrosine (1 : 1000; Cell Signaling Technology [CST]), ZO-1 (1 : 500; CST), occludin (1 : 500; CST), TNF-*α* (1 : 1000; CST), IL-*β* (1 : 1000; CST), and GAPDH (1 : 1000; CST). Next, an IRDye800CW-conjugated secondary antibody was used to probe blots, after which an Odyssey imaging system (LICOR) was used to detect protein bands.

### 2.7. Gelatin Zymography

MMP-2 and MMP-9 activity in brain tissue homogenates was quantified via gelatin zymography as in previous studies [14]. Briefly, a total of 50 *μ*g of a given lysate sample was added to a well holding 10% acrylamide gels containing 0.1% gelatin. Tris-glycine running buffer was then used for separation, after which gels were washed and added to renaturing buffer for 1 h at 37°C. Next, gels were added to the development buffer for 24 h at 37°C, after which they were stained for 1 h using 0.5% Coomassie Blue G-250. Destaining solution was then used to treat gels thrice, after which ImageJ was used to quantify MMP activity.

### 2.8. Statistical Analyses

Data are means ± SEM. Samples were compared via two-way ANOVAs with Tukey's post hoc test, with *p* < 0.05 as the significance threshold.

## 3. Results

### 3.1. Blood Glucose Level Measurement

**Figure 1:**
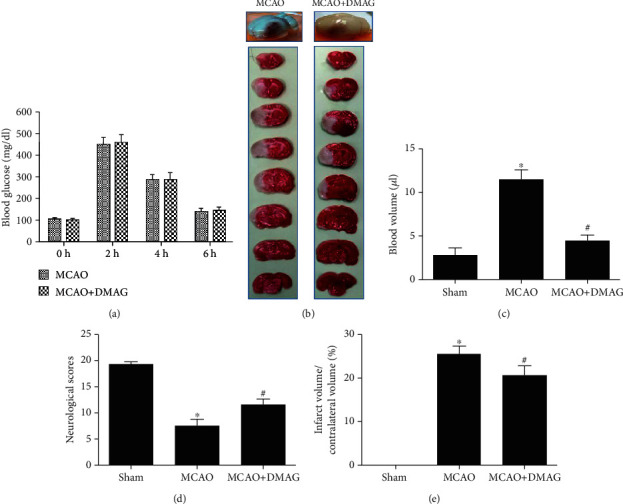
The effects of 17-DMAG on HT and neurological function in hyperglycemic MCAO rats. (a) 17-DMAG had no effects on blood glucose at the 4 time points. (b) Representative pictures of whole brain and TTC stained slices. 17-DMAG reduced hemorrhagic transformation following MCAO and decreased infarction volume. (c) Statistical analysis of blood volume in each group. 17-DMAG significantly reduced hyperglycemia-enhanced hemorrhagic volume at 24 h after MCAO. (d) Neurological function scoring at 24 h after MCAO. 17-DMAG significantly improved the neurological deficit at 24 h. ^∗^*p* < 0.05 compared with sham group; ^#^*p* < 0.05 compared with MCAO group. *n* = 5‐8 for each group.

At 2 h postdextrose injection, injected animals had significantly increased blood glucose levels relative to at baseline, and these levels remained elevated for up to 6 h. No impact on blood glucose levels was observed following 17-DMAG treatment (Figure 1(a)) Moreover, treatment of mice with 0.2 mg/kg 17-DMAG barely affected the body weight of the mice, suggesting the possible safety of 17-DMAG at this low dose.

### 3.2. 17-DMAG Modulates Hyperglycemia-Enhanced HT following MCAO

Consistent with past reports, we found that hyperglycemia led to extensive HT in ischemic areas of the brain in MCAO model rats at 24 h post-MCAO. Relative to animals injected with vehicle control, animals administered 17-DMAG exhibited significant reductions in both infarct size and hemorrhage volume at 24 h post-MCAO (Figures 1(b)–1(d)). There was also a clear correlation between infarct size and hemorrhage volume in these animals (Figures 1(b)–1(d)). Relative to sham controls, MCAO model animals exhibited clear neurological deficits that were partially reversed in MCAO model animals that had been treated with 17-DMAG (Figure 1(e)).

### 3.3. 17-DMAG Treatment Suppresses MCAO-Mediated Changes in Tight Junction Protein Levels

**Figure 2:**
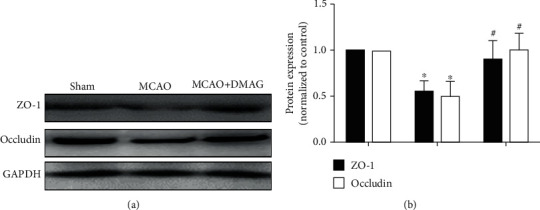
Effects of 17-DMAG on tight junction proteins in hyperglycemic MCAO rats. (a) Representative Western blots of ZO-1 and occludin. (b) 17-DMAG significantly restored occludin and ZO-1 downregulation. ^∗^*p* < 0.05, compared with the sham group; ^#^*p* < 0.05, compared with the MCAO group; *n* = 3 for each group.

We next assessed whether 17-DMAG modulates BBB disruption in hyperglycemic MCAO model rats via measuring levels of the tight junction proteins occludin and ZO-1 via Western blotting. We observed significant reductions in occludin and ZO-1 levels in MCAO model animals, whereas 17-DMAG treatment significantly reduced these MCAO-associated changes (Figure 2).

### 3.4. 17-DMAG Alters MMP Activity in Hyperglycemic MCAO Model Rats

**Figure 3:**
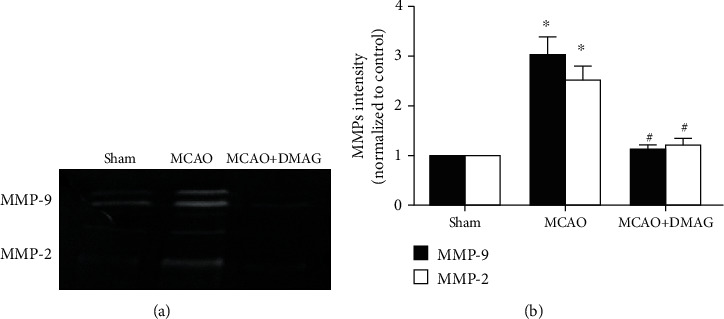
Effects of 17-DMAG on MMP activation in hyperglycemic rats after MCAO. (a) Representative bans for MMP-9 and MMP-2. (b) Statistical analysis for MMP-9 and MMP-2 activation. 17-DMAG remarkably decreased the activity of MMP-9 and MMP-2. ^∗^*p* < 0.05, compared with the sham group; ^#^*p* < 0.05, compared with the MCAO group; *n* = 3 for each group.

We next extended our analysis of how 17-DMAG impacts HT following MCAO in hyperglycemic rats through an assessment of MMP-2 and MMP-9 activity via a gelatin zymography approach. We observed significant increases in MMP-2 and MMP-9 activity at 24 h post-MCAO in hyperglycemic animals, whereas 17-DMAG treatment significantly reduced the activity of these MMPs (Figure 3). This thus suggests that 17-DMAG can suppress hyperglycemia-induced HT in part via inhibiting MMP-2 and MMP-9 following MCAO.

### 3.5. 17-DMAG Modulates Oxidative Stress in Hyperglycemic MCAO Model Rats

**Figure 4:**
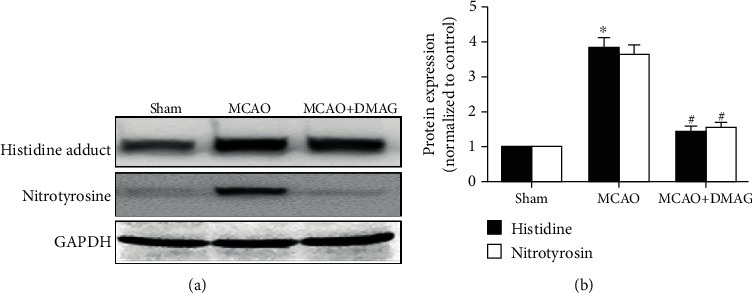
Effects of 17-DMAG on oxidative stress in hyperglycemic rats after MCAO. (a) Representative immunoblots of histidine adduct and nitrotyrosine at 24 h in the ischemic hemisphere. (b) Statistical analysis for the Western blot showed increased histidine adduct and nitrotyrosine levels in the MCAO group which were restored by 17-DMAG. ^∗^*p* < 0.05, compared with the sham group; ^#^*p* < 0.05, compared with the MCAO group; *n* = 3‐4 for each group.

We observed significant increases in MDA content in ischemic brain tissues at 24 h post-MCAO, whereas these levels were significantly lower in animals treated with 17-DMAG. Consistent with this result, we similarly observed increased 4-hydroxy-2-nonenal and nitrotyrosine levels in MCAO model animals at 24 h post-MCAO, with 17-DMAG reducing these levels (Figure 4).

### 3.6. 17-DMAG Suppresses Inflammation in Hyperglycemic MCAO Model Rats

**Figure 5:**
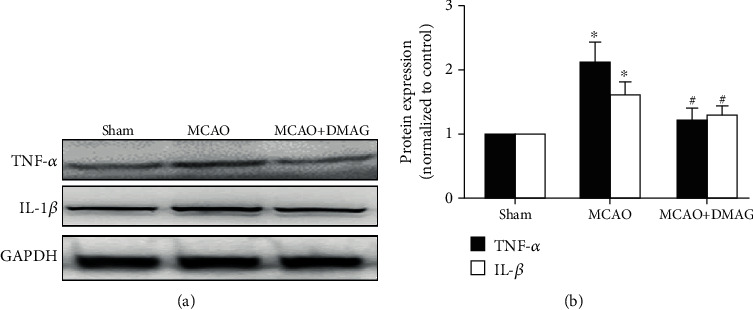
Effects of 17-DMAG on inflammation in hyperglycemic rats after MCAO. (a) Representative Western blots of TNF-*α* and IL-1*β*. (b) 17-DMAG effectively inhibited the increase of TNF-*α* and IL-1*β* in hyperglycemic rats after MCAO. ^∗^*p* < 0.05, compared with the sham group; ^#^*p* < 0.05, compared with the MCAO group; *n* = 3 for each group.

At 24 h post-MCAO, rats exhibited significant increases in TNF-*α* and IL-1*β* levels, whereas 17-DMAG suppressed the upregulation of these inflammatory cytokines (Figure 5).

## 4. Discussion/Conclusion

The present report expands upon our previous study by demonstrating that the Hsp90 inhibitor 17-DMAG can both reduce brain infarction and suppress hyperglycemia-enhanced HT following MCAO in rats. Our results further suggest that 17-DMAG can prevent HT at least in part by decreasing inflammatory TNF-*α* and IL-1*β* production, suppressing oxidative stress, and inhibiting MMP-9 activity. As such, 17-DMAG may offer value as a therapeutic compound that can prevent HT in patients suffering from ischemic stroke.

Hsp90 functions as a key chaperone that supports the folding of many essential proteins within cells, including signaling proteins and transcription factors [15]. The Hsp90 inhibitor 17-DMAG reportedly suppresses inflammation and oxidative stress in many different disease models, including models of ischemic stroke and abdominal aortic aneurysm. We have previously shown that acute stroke patients exhibit increased expression of both serum Hsp90 and MMP-9, suggesting that Hsp90 may thus be related to BBB disruption in patients suffering from stroke. In an animal MCAO model, inhibition of Hsp90 has been shown to mediate neuroprotection that is associated with reduced inflammation and oxidative stress, as well as with reduced MMP activity and enhanced BBB integrity. Elevated MMP expression and activity has been shown to play a central role in stroke-associated breakdown of the BBB owing to the degradation of tight junctions [16]. Disruption of the BBB is associated with increased vascular permeability, resulting in more blood cells entering the parenchyma, thus driving hemorrhagic transformation. Up to 40% of ischemic stroke patients are hyperglycemic due to either acute stress or diabetes upon hospitalization. In the present study, we therefore explored the impact of the Hsp90 inhibitor 17-DMAG on hyperglycemia-induced HT in a rat MCAO model system. Consistent with previous reports, we found that 17-DMAG treatment enhanced neurological functionality and reduced HT in these animals, with improved tight junction integrity and MMP inhibition. In the mechanism, similar to our previous research in the bEnd.3 cell, it is speculated that 17-DMAG may inhibit MMP transcription and secretion by disrupting the binding of Hsp90 and IKK, which may lead to the degradation of IKK and subsequent inhibition of the binding of P65 to the promoter regions of MMP-9 [8]. Of note, 17-DMAG was also found to inhibit ANG II-induced expression of MMP-2 and MMP-9 via inhibition of AP-1 transcriptional activity [7].

HT is a serious event that occurs during reperfusion in hyperglycemic patients. Such injury is most often associated with oxidative stress and cell membrane lipid damage, resulting in cellular death. Hyperglycemia leads to increased production of reactive oxygen species (ROS) and associated ROS-mediated signaling, thus increasing levels of neuronal damage [17–19]. Importantly, there is substantial evidence suggesting that Hsp90 is a key regulator of ROS generation owing to its ability to interact with enzymes in the nicotinamide adenine dinucleotide phosphate oxidase (Nox) family which mediates ROS production [19–20]. We have previously shown that 17-DMAG-mediated inhibition of Hsp90 can improve ROS scavenging and thereby improve ischemia/reperfusion injury outcomes. In this study, we determined that, consistent with previous findings, hyperglycemia and ischemia/reperfusion injury were associated with significant oxidative stress, potentially leading to BBB disruption and cerebral edema, with 17-DMAG being sufficient to suppress this damage associated with hyperglycemia-induced HT.

Inflammation is well-known to be closely associated with the pathogenesis of ischemic injuries. Hsp90 has also been shown to be a key regulator of inflammation, making it an important target of therapeutic intervention in patients suffering from ischemic stroke. We have previously shown that the Hsp90 inhibitor 17-DMAG can reduce damaging inflammation and oxidative stress in an animal model of stroke, thus suggesting that 17-DMAG or other Hsp90 inhibitors may be ideal for achieving neuroprotection in the context of cerebral ischemia [8]. Other studies have shown that Hsp90 inhibition can reduce the upregulation of inflammatory cytokines in ischemic brain tissue via disruption of IKK activity and associated NF-*κ*B activation [21]. Consistent with these past findings, in the present report, we found that 17-DMAG suppressed inflammation and MMP-9 activity, thereby preventing BBB disruption and HT development in an animal model of ischemic stroke.

In conclusion, we demonstrated that 17-DMAG, an HSP90 inhibitor, markedly attenuated hyperglycemia-enhanced HT and improved neurological function after MCAO through simultaneously inhibiting ROS production, MMP expression, and inflammatory response. Although 17-DMAG has been withdrawn from the clinical trials for the treatment of cancer patients due to side effects, today, clinical trials of tens of Hsp90 inhibitors in oncological indications are underway [9]. Therefore, our findings may have direct translational implications for HT following ischemia stroke.

## Data Availability

The data used to support the findings of this study are available from Dr. Feng Wang upon request.
